# Is epirubicin effective in first-line chemotherapy of metastatic breast cancer (MBC) after an epirubicin-containing adjuvant treatment? A single centre phase III trial

**DOI:** 10.1038/sj.bjc.6603096

**Published:** 2006-04-18

**Authors:** C Pacilio, A Morabito, F Nuzzo, A Gravina, V Labonia, G Landi, E Rossi, E De Maio, M Di Maio, G D'aiuto, G Botti, N Normanno, P Chiodini, C Gallo, F Perrone, A de Matteis

**Affiliations:** 1 Medical Oncology C, National Cancer Institute, Via Mariano Semmola, 80131 Naples, Italy; 2 Clinical Trials Unit, National Cancer Institute, Via Mariano Semmola, 80131 Naples, Italy; 3 Senology, National Cancer Institute, Via Mariano Semmola, 80131 Naples, Italy; 4 Pathology, National Cancer Institute, Via Mariano Semmola, 80131 Naples, Italy; 5 Cell Biology and Preclinical Models, National Cancer Institute, Via Mariano Semmola, 80131 Naples, Italy; 6 Medical Statistics, Second University, Naples, Italy

**Keywords:** epirubicin, docetaxel, metastatic breast cancer, randomised trials, adjuvant anthracyclines

## Abstract

The aim of the study was to demonstrate the superiority of docetaxel and epirubicin *vs* docetaxel alone as first-line therapy in metastatic breast cancer patients pretreated with adjuvant or neoadjuvant epirubicin. We compared single agent docetaxel 100 mg m^−2^ (D) with the combination of docetaxel 80 mg m^−2^ and epirubicin 75 mg m^−2^ (ED). The response rate (72 *vs* 79%), the progression-free survival (median 9 *vs* 11 months) and the overall survival (median 18 *vs* 21 months) were not significantly different between the ED (*n*=26) and D arms (*n*=25), respectively. Leucopaenia, nausea and stomatitis were significantly worse with ED. In conclusion, epirubicin should not be administered in combination with taxanes in metastatic breast cancer patients relapsed after an anthracycline-based adjuvant or neoadjuvant therapy.

Anthracyclines are among the most active agents in the treatment of metastatic and adjuvant breast cancer (Fossati *et al*, 1998; EBCTCG, 2004). Docetaxel is the only agent that has demonstrated survival benefits in anthracycline-resistant patients (Nabholtz *et al*, 1999) and superior activity over doxorubicin as first-line chemotherapy in patients previously treated with alkylating agents (Chan *et al*, 1999). Owing to its activity and to the lack of cross-resistance with anthracyclines, there is a strong rationale for combining docetaxel with anthracyclines. The anthracycline-taxane regimens can now be considered the most effective regimens in metastatic breast cancer (MBC) and a first-line therapy of choice in this set of patients (Valero and Hortobagyi, 2003). In particular, many reports have described the combined use of docetaxel and epirubicin, an anthracycline less cardiotoxic than doxorubicin, with the objective to find the safest and most efficient way to integrate these classes of drugs (Mavroudis *et al*, 2000; Pagani *et al*, 2000). However, whether an anthracycline-taxane regimen is worthy as first-line treatment for MBC patients pretreated with adjuvant or neoadjuvant anthracyclines is an open question, not definitely assessed by appropriate prospective trials. Therefore, we planned a randomised phase III trial to compare epirubicin and docetaxel *vs* docetaxel alone for efficacy and safety as first-line chemotherapy of MBC patients pretreated with epirubicin in adjuvant or neoadjuvant setting.

## MATERIALS AND METHODS

Eligibility criteria were: women with MBC⩽65 years; no previous chemotherapy for metastatic disease; measurable disease; Eastern Cooperative Oncology Group (ECOG) performance status of 0–2; previous adjuvant or neoadjuvant chemotherapy with anthracyclines, up to a total dose of doxorubicin ⩽250 mg m^−2^ or epirubicin ⩽500 mg m^−2^, completed at least 12 months before enrollment; absence of brain metastases; adequate bone marrow, renal and liver function; left ventricular ejection fraction (LVEF) ⩾50%. Previous adjuvant endocrine therapy as well as endocrine therapy for metastatic disease were allowed. Patients were excluded if they had received taxanes as adjuvant chemotherapy or had history of serious medical conditions potentially compromising study participation. Pregnant or lactating women were ineligible. All patients were required to provide written informed consent and the protocol was approved by the Independent Ethical Committee of the National Cancer Institute of Naples.

Patients were randomly assigned to docetaxel 100 mg m^−2^ (Arm D) or to epirubicin 75 mg m^−2^ and docetaxel 80 mg m^−2^ (Arm ED) on day 1, every 3 weeks for six cycles of chemotherapy, unless disease progression or unacceptable toxicity. Prednisone and a prophylactic antiemetic regimen with 5-HT3 antagonists were given from the first infusion. No dose reduction of chemotherapy was planned by protocol. Treatment was delayed for 1 week for grade ⩾2 neutropenia and/or grade ⩾1 thrombocytopenia. Granulocyte colony-stimulating factor (G-CSF) was administered at 5 mcg kg day^−1^ subcutaneously in case of grade 4 neutropenia until neutrophil count >2000 mm^−3^.

Pretreatment evaluation, performed within 1 month before randomisation, included physical examination, laboratory studies, ECG, echocardiography with LVEF, brain, chest and abdomen computed tomography, bone scan, skeletal radiographs (if required). Echocardiography and evaluation of tumour response, according to Response evaluation criteria in solid tumor (RECIST) guidelines, were performed every three cycles.

Toxicity was evaluated according to National Cancer Institute Common Toxicity Criteria version 2.0. For each type of toxicity, the worst degree experienced throughout the treatment was computed for each patient.

The study was designed as a single centre randomised phase III study with response rate as primary end point. Stratification factors were: performance status (0 *vs* 1 *vs* 2), disease-free survival (<24 months *vs* >24 months) and type of dominant metastatic site (soft tissue *vs* bone *vs* viscera). Primary objective was to evaluate the objective response rate (ORR) of docetaxel and epirubicin compared with docetaxel alone. Secondary end points included toxicity, progression-free survival (PFS) and overall survival. Calculation of sample size was based on a 40% expected response rate with docetaxel alone, a 60% auspicated response rate with the combination of docetaxel and doxorubicin, a 20% of risk of false negative and a 5% of risk of false positive result, and a one-tailed *χ*^2^ test. With these requirements, planned sample size was 77 patients for each arm of treatment. Continuous and ordinal categorical data were compared by exact Wilcoxon rank sum test. Dichotomous data were compared by Fisher's exact test. Progression-free and overall survival curves were drawn according to Kaplan–Meier and statistical significance of differences was tested by the log-rank test. As the study was interrupted earlier than planned, the Bayesian predictive probability that the response rate of the ED group would be statistically better than that of the D group, if the trial were continued to the planned end, was calculated to corroborate the meaning of findings (Johns and Andersen, 1999).

## RESULTS

From May 2000 to October 2003, 51 patients were enrolled. The recruitment was slower than expected, due to the restrictive inclusion criteria of the protocol and prompted us to stop the study, after 3.5 years, because time needed to reach the planned sample size would have been too long. Baseline characteristics of the patients were well balanced between the two arms (Table 1).

The median number of administered cycles was six (range 2–6); 81 and 76% of patients received all the planned therapy (six cycles) in the ED and D treatment arms, respectively. Median average relative dose intensity of treatment was similar in the two groups, while G-CSF was used far more frequently in ED group; 77% of ED subjects, indeed, used G-CSF in at least one cycle compared with 56% of D patients (*P*=0.0582).

All the responses were independently validated. Both therapeutic regimens showed similar antitumour activity (Table 2). The ORR was 72% (18 patients; 95% exact CI: 51–88) in the ED arm and 79% (19 patients; 95% exact CI: 58–93) in the D arm (one-tailed *P*=0.8196). Based on these results, the probability that the response rate of the ED group would be significantly better than that of the D group, if the trial were brought to its completion, is equal to 0.0334.

**Figure 1:**
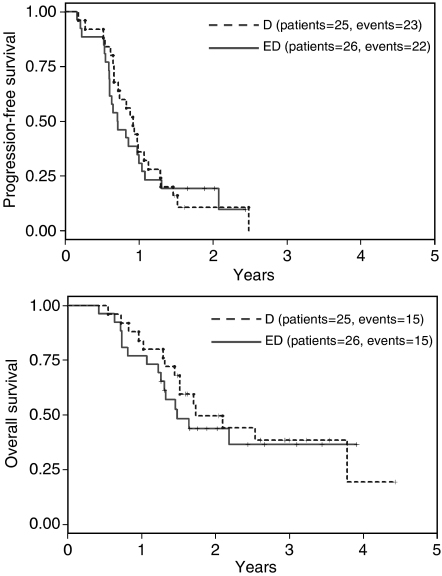
Kaplan–Meier estimated progression-free (top) and overall survival (bottom) curves scattered by treatment arm (D=blue dotted line; ED=red solid line).

After 45 (88%) events, the median progression-free survival was 9 months in the ED and 11 months in the D arm (one-tailed *P*=0.6998). With regard to overall survival, 30 events (15 in each arm) were reported after a 30 months median follow-up of alive patients. The median overall survival was 18 months in the ED and 21 months in the D arm (one-tailed *P*=0.6406). Progression-free and overall survival curves are reported in Figure 1.

Haematological and nonhaematological toxicities are summarised in Table 3. Leucopaenia, the most frequent haematological toxicity, was significantly more severe in the ED arm (*P*=0.0290). Among nonhaematological toxicities, nausea and stomatitis were significantly worse in the ED arm (*P*=0.0210 and *P*=0.0499, respectively). No significant differences between the arms were found in other nonhaematological toxicities. Grade 1 cardiac toxicity was reported in 19 and 16% of patients, with ED and D, respectively (*P*=0.9999).

## DISCUSSION

Our phase III study indicates that the addition of epirubicin to docetaxel did not improve outcomes as compared to single-agent docetaxel, in the first-line treatment of metastatic breast cancer patients, who already had received adjuvant epirubicin. Both therapeutic regimens showed similar antitumour activity, and no significant differences were found between the two treatments in progression-free survival and overall survival. Of course, we are aware that the small sample size and the fact that enrollment was stopped before than planned are major limitations of our study. However, our results are strengthened by the finding that the Bayesian predictive probability that the study hypothesis (that ED could increase by a 20% the response rate as compared with D alone) could be eventually demonstrated if the study had reached the planned sample size is definitely low (only 3.3%). To the best of our knowledge, there is no randomised study addressing the role of anthracyclines in the treatment of patients relapsing after anthracycline-based adjuvant chemotherapy. Retrospective studies, indirectly addressing this issue, have produced conflicting results. Chauvin *et al* (1990) demonstrated a low activity of the CEF regimen in patients previously treated with anthracycline-based adjuvant chemotherapy. Other authors (Venturini *et al*, 1996; Pierga *et al*, 2001) found that previous adjuvant chemotherapy could adversely affect the prognosis of MBC patients treated with an anthracycline-based first-line chemotherapy, but this effect was independent of whether adjuvant chemotherapy was CMF- or anthracycline-based. On the contrary, other studies (Buzdar *et al*, 1981; Valagussa *et al*, 1986; Kardinal *et al*, 1988; Gennari *et al*, 2004) did not demonstrate a poorer outcome in metastatic breast cancer patients previously treated with adjuvant chemotherapy. Namely, Gennari *et al* (2004) did not find any negative influence of adjuvant anthracyclines on the activity of first-line epirubicin and paclitaxel, confirming that modern chemotherapy regimens, including anthracyclines and taxanes, provide satisfactory results in metastatic breast cancer patients, regardless of previous adjuvant chemotherapy.

In conclusion, the results of our trial support that epirubicin should not be included in taxanes-containing regimens for breast cancer patients relapsed after an anthracycline-based adjuvant or neoadjuvant therapy, also taking into account the availability for these patients of new active, non-cross-resistant drugs.

## CONFLICT OF INTEREST

We declare no conflicts of interest.

## Figures and Tables

**Table 1:** Baseline characteristics of patients

**Variable**	**Overall (*N*=51)**	**ED (*N*=26)**	**D (*N*=25)**
Age, median (range)	51 (35–64)	51 (38–64)	51 (35–63)
*Menopausal status, n (%)*			
Premenopausal	9 (18)	5 (19)	4 (16)
Postmenopausal	42 (82)	21 (81)	21 (84)
			
*Performance status, n (%)*			
0	32 (63)	16 (62)	16 (64)
1	18 (35	9 (35)	9 (36)
2	1 (2)	1 (4)	—
			
Disease-free interval, median (range) months	31 (8–138)	33 (8–138)	30 (11–78)
*Disease-free interval, n (%)*			
⩽2 years	11 (22)	6 (23)	5 (20)
>2 years	40 (78)	20 (77)	20 (80)
			
*Dominant metastatic site, n (%)*			
Soft tissues	21 (41)	12 (46)	9 (36)
Bone	1 (2)	—	1 (4)
Viscera	29 (57)	14 (54)	15 (60)
			
*Previous radiation following conservative surgery*			
No	30 (59)	17 (65)	13 (52)
Yes	21 (41)	9 (35)	12 (48)
			
Previous dose of anthracycline, median (range), (mg m^−2^)	440 (150–500)	440 (150–480)	440 (300–500)
Time from last anthracycline, median (range), (months)	37 (12–131)	35 (12–131)	37 (18–83)

**Table 2:** Outcomes

**Variable**	**Overall (*N*=51)**	**ED (*N*=26)**	**D (*N*=25)**	***P* a**
*Objective response analysis, n (%)*				
Not eligible	2 (4)	1 (4)	1 (4)	
Eligible	49 (96)	25 (96)	24 (96)	
Best response, *n* (% of eligible patients)				
Complete	10 (20)	4 (16)	6 (25)	
Partial	27 (55)	14 (56)	13 (54)	
Stable disease	7 (14)	4 (16)	3 (12)	
Progressive disease,	4 (8)	3 (12)	1 (4)	
Not assessed	1 (2)	—	1 (4)	
Objective response rate, % (95% exact CI)	76 (61–87)	72 (51–88)	79 (58–93)	0.8196^b^
				
Time to progression (TTP)				0.6998^c^
Events, *n* (%)	45 (88)	22 (85)	23 (92)	
Median (95% confidence interval) TTP, (months)	10 (8–12)	9 (7–13)	11 (9–15)	
				
Overall survival				0.6406^c^
Events, *n* (%)	30	15	15	
Median (95% confidence interval) overall survival, (months)	20 (17-na)	18 (15-na)	21 (18-na)	

a One-tailed *P*-values.

b From Fisher exact test.

c From Log-rank test.

**Table 3:** Worst per-patient toxicity according to NCI-CTC grade^a^

	**ED (*N*=26)**					**D (*N*=25)**					
**Type of toxicity**	**0**	**1**	**2**	**3**	**4**	**0**	**1**	**2**	**3**	**4**	***P* b**
Allergy	96	—	4	—	—	92	4	4	—	—	0.9700
Anemia	62	27	4	8	—	72	28	—	—	—	0.3315
Leukopenia	15	—	12	38	35	20	—	28	48	4	**0.0290**
Neutropenia	12	—	4	8	77	20	—	4	16	60	0.2453
Febbrile neutropenia	88	—	—	12	—	92	—	—	4	4	0.9999
Platelets	96	4	—	—	—	100	—	—	—	—	0.9999
Heart	81	19	—	—	—	84	16	—	—	—	0.9999
Fatigue	50	27	19	4	—	60	24	16	—	—	0.4210
Fever	85	15	—	—	—	84	16	—	—	—	0.9999
Weight loss	96	—	4	—	—	100	—	—	—	—	0.9999
Hair loss	27	—	73			24	—	76			0.9999
Skin	92	8	—	—	—	88	4	8	—	—	0.5162
Anorexia	77	23	—	—	—	96	4	—	—	—	0.1108
Constipation	92	8	—	—	—	92	4	4	—	—	0.9700
Diarrhoea	46	23	27	4	—	28	36	32	4	—	0.3320
Nausea	27	46	23	4	—	60	28	12	—	—	**0.0210**
Stomatitis	31	42	15	12	—	56	32	12	—	—	**0.0499**
Vomiting	58	27	15	—	—	80	16	4	—	—	0.0968
Liver	96	—	—	4	—	96	—	—	4	—	0.9999
Motor neuropathy	92	8	—	—	—	92	8	—	—	—	0.9999
Sensorial neuropathy	88	12	—	—	—	84	16	—	—	—	0.9543
DIC	96	—	—	4	—	100	—	—	—	—	0.9999
RBC transfusion	96	—	—	4	—	100	—	—	—	—	0.9999

a Row percentages are reported; sum can be different from 100 because of rounding.

b Two-tailed *P*-values from exact Wilcoxon–Mann–Whitney test. Bold values are statistically significant.

## **Appendix A1**

Members of the NCI-Naples Breast Cancer Group who contributed to this manuscript:

Cell Biology and Preclinical Models Unit: Nicola Normanno, Antonella De Luca, Monica Rosaria Maiello, Adele Carotenuto.

Clinical Trials Unit: Francesco Perrone, Alessandro Morabito, Ermelinda De Maio, Massimo Di Maio, Roberta D’Aniello, Gianfranco De Feo.

Medical Oncology C: Andrea de Matteis, Francesca Di Rella, Adriano Gravina, Vincenzo Labonia, Gabriella Landi, Francesco Nuzzo, Carmen Pacilio, Emanuela Rossi.

Nuclear Medicine: Secondo Lastoria, Aldo Bartiromo.

Pathology: Gerardo Botti, Maurizio Di Bonito, Franca La Vecchia, Maria Staiano, Franca Formichelli, Maria Pia Curcio.

Pharmacy: Maria Rosaria Salzano.

Radiotherapy: Brunello Morrica.

Senology: Giuseppe D’Aiuto, Franca Avino, Immacolata Capasso, Claudio Longo, Michele Pizzorusso, Massimo Rinaldo, Renato Thomas.
